# Histopathological growth patterns as biomarker for adjuvant systemic chemotherapy in patients with resected colorectal liver metastases

**DOI:** 10.1007/s10585-020-10048-w

**Published:** 2020-07-20

**Authors:** Florian E. Buisman, Eric P. van der Stok, Boris Galjart, Peter B. Vermeulen, Vinod P. Balachandran, Robert R. J. Coebergh van den Braak, John M. Creasy, Diederik J. Höppener, William R. Jarnagin, T. Peter Kingham, Pieter M. H. Nierop, Eran Sadot, Jinru Shia, Bas Groot Koerkamp, Dirk J. Grünhagen, Michael D’Angelica, Cornelis Verhoef

**Affiliations:** 1 Department of Surgery, Erasmus MC Cancer Institute, Dr. Molewaterplein 40, 3015 GD Rotterdam, The Netherlands; 2 Department of Surgery, Memorial Sloan Kettering Cancer Center, New York, USA; 3 Department of Oncological Research, Oncology Center, GZA Hospitals Campus Sint-Augustinus and University of Antwerp, Antwerp, Belgium; 4 Department of Pathology, Memorial Sloan Kettering Cancer Center, New York, USA

**Keywords:** Colorectal cancer, Colorectal liver metastases, Histopathological growth pattern, Chemotherapy

## Abstract

Adjuvant systemic chemotherapy (CTx) is widely administered in patients with colorectal liver metastases (CRLM). Histopathological growth patterns (HGPs) are an independent prognostic factor for survival after complete resection. This study evaluates whether HGPs can predict the effectiveness of adjuvant CTx in patients with resected CRLM. Two main types of HGPs can be distinguished; the desmoplastic type and the non-desmoplastic type. Uni- and multivariable analyses for overall survival (OS) and disease-free survival (DFS) were performed, in both patients treated with and without preoperative chemotherapy. A total of 1236 patients from two tertiary centers (Memorial Sloan Kettering Cancer Center, New York, USA; Erasmus MC Cancer Institute, Rotterdam, The Netherlands) were included (period 2000–2016). A total of 656 patients (53.1%) patients received preoperative chemotherapy. Adjuvant CTx was only associated with a superior OS in non-desmoplastic patients that had not been pretreated (adjusted hazard ratio (HR) 0.52, 95% confidence interval (CI) 0.37–0.73, p < 0.001), and not in desmoplastic patients (adjusted HR 1.78, 95% CI 0.75–4.21, p = 0.19). In pretreated patients no significant effect of adjuvant CTx was observed, neither in the desmoplastic group (adjusted HR 0.83, 95% CI 0.49–1.42, p = 0.50) nor in the non-desmoplastic group (adjusted HR 0.96, 95% CI 0.71–1.29, p = 0.79). Similar results were found for DFS, with a superior DFS in non-desmoplastic patients treated with adjuvant CTx (HR 0.71, 95% CI 0.55–0.93, p < 0.001) that were not pretreated. Adjuvant CTx seems to improve OS and DFS after resection of non-desmoplastic CRLM. However, this effect was only observed in patients that were not treated with chemotherapy.

## Introduction

Pre- and or postoperative systemic chemotherapy is often administered in patients with potentially resectable colorectal liver metastases (CRLM). The effectiveness has been investigated in randomized controlled trials [1–4]. The long-term follow-up of a phase III trial demonstrated a superior early progression-free survival (PFS) for patients treated with perioperative FOLFOX. However, there was no difference in overall survival (OS) with long term follow-up [5].

Retrospective studies have suggested that the effectiveness of systemic chemotherapy may depend on the extent of disease or factors associated with OS. Potentially positive associations of perioperative systemic chemotherapy and OS were seen in populations with a high clinical risk score (CRS), or elevated preoperative carcinoembryonic antigen (CEA) levels [6–8]. In order to adequately identify subgroups that benefit from adjuvant chemotherapy (CTx) after resection of CRLM, biomarkers that reflect actual tumor biology are needed.

**Fig. 1:**
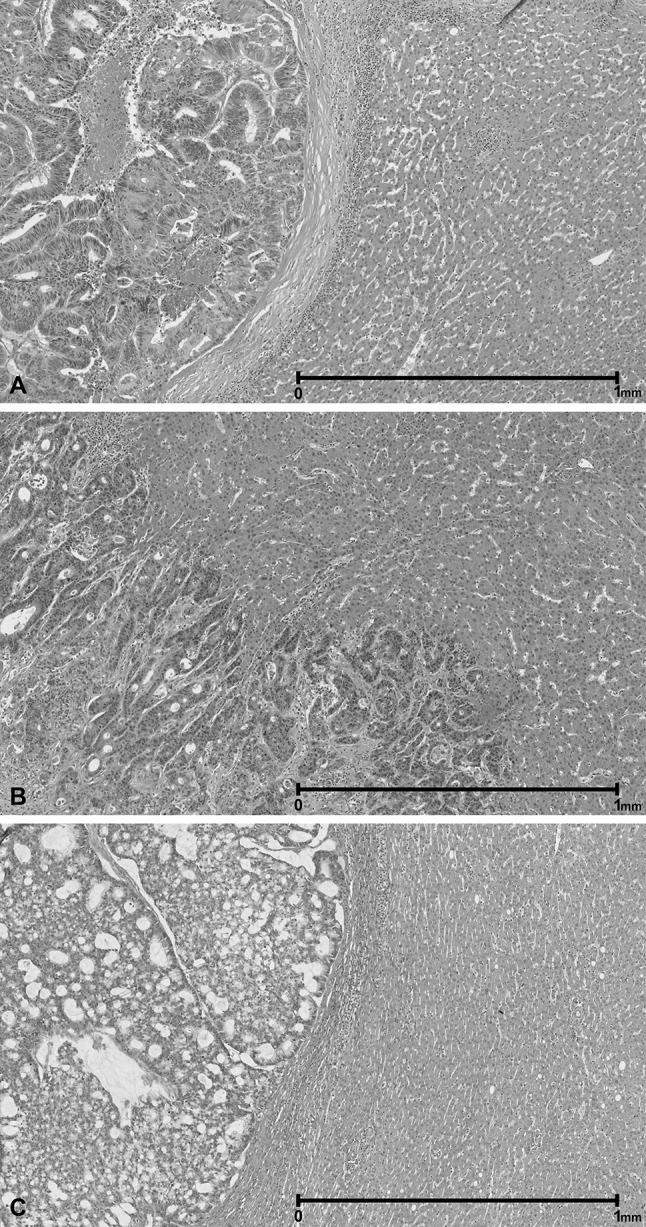
H&E images of the HGP types. H&E tissue section. **a** Desmoplastic HGP; **b** replacement HGP; **c** pushing HGP

Recent studies have suggested that the histopathological growth patterns (HGPs) of CRLM, obtained from hematoxylin and eosin (H&E) stained tissue sections after resection, are able to identify patients with an unfavorable tumor biology [9–11]. Two main types of HGPs can be distinguished; a desmoplastic type (dHGP) and a non-desmoplastic type (non-dHGP) [10, 12]. The dHGP is driven by angiogenesis and elevated infiltration of immune cells is observed. Morphologically these tumors are characterized by a desmoplastic rim surrounding the tumor border. In non-dHGP CRLM, the tumor cells replace the liver parenchyma by using pre-existing liver vessels for blood supply (i.e. vessel co-option) instead of angiogenesis [11, 12]. Non-dHGP has been associated with a worse prognosis for patients undergoing resection of CRLM in multiple studies [10, 13, 14]. A large cohort study suggested that this effect was predominantly found in patients that were not pretreated with chemotherapy prior to CRLM resection [10] (Fig. 1).

As HGPs reflect biological processes associated with tumor growth, this factor may be used to assess the effect of adjuvant CTx. This multicenter study aimed to evaluate if HGPs can be used to predict the effectiveness of adjuvant CTx after resection of CRLM.

## Methods

### Study population

**Fig. 2:**
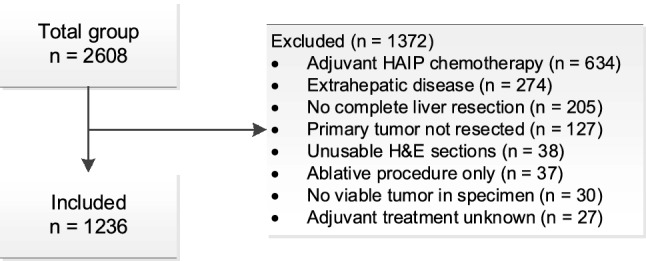
Study flowchart. HAIP: hepatic arterial infusion pump, H&E: hematoxylin and eosin

All consecutive patients who underwent a complete resection of CRLM from 2000 to 2016 at Memorial Sloan Kettering Cancer Center (MSKCC, New York, United States) and at the Erasmus MC Cancer Institute (Erasmus MC, Rotterdam, The Netherlands), were evaluated for inclusion. A total of 2608 consecutive patients were evaluated for inclusion. Patients were excluded from analysis for the following reasons: adjuvant hepatic artery infusion pump chemotherapy, R2 resection, no resection of primary tumor, extrahepatic disease prior to or at time of liver resection, and H&E stained tissue sections that were not suitable for scoring HGPs. H&E tissue sections were considered non-suitable if there was less than a 20% of the expected tumor-liver interface, showed poor tissue preservation or when viable tumor tissue was absent [13]. In total 1236 (47.4%) were eligible for inclusion (Fig. 2).

### HGP characterization

HGPs were evaluated according to international guidelines [13]. In order to determine HGP type, all available H&E stained tissue sections off all available CRLM were evaluated using light microscopy for each patient. The entire interface between tumor and adjacent liver tissue was evaluated for the type of HGP and the proportion of each HGP was scored using percentages. Average HGP percentages were calculated per metastasis and per patient (in case of multiple CRLM). This method has been validated previously, demonstrating a 95% within CRLM concordance (in case of multiple H&E slides) and a 90% between metastases concordance (in case of multiple CRLM in one patient) [14]. Patients were classified in two groups: dHGP if all available slides showed a 100% desmoplastic interface and non-dHGP if a replacement or pushing type HGP was found on one or more slides [10]. Non-dHGP CRLM represent a mix of different interfaces with a varying degree of desmoplastic, replacement, and pushing type HGPs. Pushing type HGP CRLM are rare and are vascularized by angiogenensis in the absence of a desmoplastic stromal rim [11, 12].

### Timing of chemotherapy

In MSKCC, most patients received pre- and/or postoperative (i.e. adjuvant) chemotherapy. In the Erasmus MC cohort, preoperative chemotherapy was regularly administered in referring hospitals or in patients with borderline resectable CRLM. Patients with upfront resectable CRLM were not treated with preoperative chemotherapy at Erasmus MC. Adjuvant chemotherapy is not the standard of care after resection of CRLM according to the Dutch guidelines. All analyses were performed separately for patients treated with and without preoperative chemotherapy according to the findings by Galjart et al., demonstrating limited prognostic value of HGPs in pretreated patients [10].

### Definitions

Clinicopathological data and postoperative treatment data were available from prospectively maintained databases. Synchronous CRLM were defined as detected within 3 months after resection of the primary tumor. Number and size of CRLM were derived from pathology reports. Any lesions treated with ablative therapies (Radio Frequency Ablation or Microwave Ablation) were added to the total number of CRLM treated. The clinical risk score (CRS) was calculated by assigning one point for the presence of each of the five components: node positive primary tumor, disease-free interval between resection primary and diagnosis of CRLM less than 12 months, more than one CRLM, size of largest CRLM above 5 cm, and preoperative serum carcinoembryonic antigen (CEA) level of more than 200 µg/L [8]. The CRS was subdivided into low-risk (0–2 points) and high-risk (3–5 points). A positive resection margin was defined as the presence of viable tumor at the resection margin. Preoperative chemotherapy was defined as any chemotherapy administered within six months before liver resection. Adjuvant chemotherapy was defined as any systemic chemotherapy administered within six months after liver resection as long as it was not used for recurrent disease.

### Statistical analysis

Differences between groups in baseline characteristics were evaluated using the Chi-square test for categorical variables and the Mann–Whitney U-test for continuous variables. Median follow-up time for survivors was estimated using the reversed Kaplan–Meier method. Complete case analysis for the regression analyses was performed. Survival was estimated by the Kaplan–Meier method and groups were compared using the log-rank test. OS was defined from the date of CRLM resection until the date of last follow-up or death. Disease-free survival (DFS) was defined from the date of CRLM resection until the date of recurrence, last follow-up or death. Uni- and multivariable analyses of OS and DFS were performed with Cox proportional hazard modeling. Results were reported as hazard ratios (HR) with 95% confidence intervals (CI). A p-value of less than 0.05 was considered statistically significant. Analyses were performed using SPSS (IBM Corp, version 24, Armonk, NY) and RStudio (RStudio, version 1.0.153, Boston, MA; survival package).

## Results

### Patient characteristics

A comparison at baseline was made between patients treated with and without adjuvant CTx (Table 1). Patients that were not pretreated who received adjuvant CTx had more common left-sided primary tumors (50.0% versus 40.4%, p < 0.001). Patients that were pretreated who received adjuvant CTx had more advanced T-stage (pT3-4) primaries (91.5% versus 84.6%, p = 0.03).

**Table 1:** Baseline characteristics (n = 1236)

	Not pretreated				Pretreated			
	All patients	No adjuvant CTx	Adjuvant CTx	P value	All patients	No adjuvant CTx	Adjuvant CTx	P value
Sample size	580 (100%)	451 (77.8%)	129 (21.2%)	–	656 (100%)	488 (74.4%)	168 (25.6%)	
Age (median, IQR)	66.0 (58.0–74.0)	66.0 (59.0–74.0)	66.0 (55.0–72.0)	0.84	62.0 (53.0–69.0)	63.0 (54.0–70.0)	58.0 (49.0–66.0)	0.05
Gender				0.08				0.27
Male	358 (61.7%)	287 (63.6%)	71 (55.0%)		410 (62.5%)	311 (63.7%)	99 (58.9%)	
Female	222 (38.3%)	164 (36.4%)	58 (45.0%)		246 (37.5%)	177 (36.3%)	69 (41.1%)	
Center				< 0.001				< 0.001
MSKCC	203 (35.0%)	76 (16.9%)	127 (98.4%)		352 (53.7%)	188 (38.5%)	164 (97.6%)	
Erasmus MC	377 (65.0%)	375 (83.1%)	2 (1.6%)		304 (46.3%)	300 (61.5%)	4 (2.4%)	
Colorectal cancer								
Primary tumor location				< 0.001				0.33
Right-sided	134 (23.8%)	91 (20.8%)	43 (3.7%)		143 (22.5%)	104 (21.7%)	39 (25.0%)	
Left-sided	239 (42.5%)	177 (40.4%)	62 (50.0%)		305 (48.0%)	227 (47.3%)	305 (48.0%)	
Rectum	189 (33.6%)	170 (38.8%)	19 (15.3%)		188 (29.6%)	149 931.0%)	188 (29.6%)	
Missing	18				20			
pT-stage				0.27				0.03
T 0–2	106 (18.7%)	87 (19.7%)	19 (15.3%)		82 (13.7%)	69 (15.4%)	13 (8.5%)	
T 3–4	460 (81.3%)	355 (80.3%)	105 (84.7%)		518 (86.3%)	378 (84.6%)	140 (91.5%)	
Missing	14				56			
Nodal status primary tumor				0.86				0.98
N0	260 (45.4%)	202 (45.3%)	58 (45.7%)		226 (35.2%)	167 (35.0%)	59 (35.8%)	
N1	214 (37.3%)	165 (37.0%)	49 (38.6%)		249 (38.8%)	186 (39.0%)	63 (38.2%)	
N2	99 (17.3%)	79 (17.7%)	20 (15.7%)		167 (26.0%)	124 (26.0%)	43 (26.1%)	
Missing	7				14			
Colorectal liver metastases								
Synchronicity				0.62				0.20
Synchonous	205 (35.3%)	157 (34.8%)	48 (37.2%)		487 (74.2%)	356 (73.0%)	131 (78.0%)	
Metachronous	375 (64.7%)	294 (65.2%)	81 (62.8%)		169 (25.8%)	132 (27.0%)	37 (22.0%)	
Disease free interval				0.27				0.85
≤ 12 months	301 (52.0%)	240 (53.2%)	67 (52.3%)		547 (83.8%)	408 (83.6%)	139 (84.2%)	
> 12 months	278 (48.0%)	211 (46.8%)	61 (47.7%)		106 (16.2%)	80 (16.4%)	26 (15.8%)	
Missing	1				3			
Number CRLM				0.58				0.18
1	334 (57.9%)	257 (57.4%)	77 (59.7%)		208 (32.0%)	156 (32.4%)	52 (31.1%)	
2	123 (21.3%0	95 (21.2%)	28 (21.7%)		124 (19.1%)	101 (21.0%)	23 (13.8%)	
3	68 (11.8%)	55 (12.3%)	13 (10.1%)		87 (13.4%)	66 (13.7%)	21 (12.6%)	
4	31 (5.4%)	27 (6.0%)	4 (3.1%)		78 (12.0%)	56 (11.6%)	22 (13.2%)	
5–9	17 (2.9%)	11 (2.5%)	6 (4.7%)		134 (20.6%)	92 (19.1%)	42 (25.1%)	
≥ 10	4 (0.7%)	3 (0.7%)	3 (0.7%)		18 (2.8%)	11 (2.3%)	7 (4.2%)	
Missing	2				3			
Size largest tumor				0.30				0.49
≤ 5 cm	451 (80.0%)	352 (80.9%)	99 (76.6%)		542 (84.0%)	407 (84.7%)	135 (82.3%)	
> 5 cm	113 (20.0%)	83 (19.1%)	30 (23.3%)		103 (16.0%)	74 (15.4%)	29 (17.7%)	
Missing	16				11			
Preoperative CEA				0.81				0.84
≤ 200 µg/L	521 (94.6%)	409 (94.7%)	112 (94.1%)		546 (89.8%)	403 (90.0%)	143 (89.4%)	
> 200 µg/L	30 (5.4%)	23 (5.3%)	7 (5.9%)		62 (10.2%)	45 (10.0%)	17 (10.6%)	
Missing	29				48			
Clinical risk score				0.44				0.93
0–2	429 (76.1%)	333 (75.3%)	96 (78.7%)		311 (50.0%)	230 (49.9%)	81 (50.3%)	
3–5	135 (23.9%)	109 (24.7%)	26 (21.3%)		311 (50.0%)	231 (50.1%)	80 (49.7%)	
Missing	16				34			
Resection margin involved				0.50				0.47
Yes	69 (11.9%)	60 (13.4%)	9 (7.0%)		118 (18.0%)	91 (18.7%)	27 (16.2%)	
No	509 (88.1%)	389 (86.6%)	120 (93.0%)		536 (82.0%)	396 (81.3%)	140 (83.8%)	
Tumor ablation at time of resection				0.54				0.85
Yes	48 (8.3%)	39 (8.6%)	9 (7.0%)		204 (31.1%)	153 (31.4%)	51 (30.5%)	
No	532 (91.7%)	412 (91.4%)	120 (93.0%)		451 (68.9%)	335 (68.6%)	116 (69.5%)	
Missing	0				1			
CTx regimen (pre/postoperative)				< 0.001				0.82
Oxaliplatin/irinotecan based	85 (15.5%)	0	85 (82.5%)		579 (96.5%)	421 (96.5%)	158 (96.3%)	
5-FU based	18 (3.3%)	0	18 (17.5%)		21 (3.5%)	15 (3.4%)	6 (3.7%)	
No CTx	450 (81.4%)	450 (100%)	0					
Missing	27				56			
HGP				0.15				0.75
dHGP	91 (15.7%)	76 (16.9%)	15 (11.6%)		189 (28.8%)	139 (71.5%)	50 (29.8%)	
Non-dHGP	489 (84.3%)	375 (83.1%)	114 (88.4%)		467 (71.2%)	349 (28.5%)	118 (70.2%)	

*Erasmus MC* Erasmus Medical Center, *CEA* carcinoembryonic antigen, *cm* centimeter, *CRLM* colorectal liver metastases, *CTx* chemotherapy, *dHGP* desmoplastic type histopathological growth pattern, *HGP* histopathological growth pattern, *IQR* inter quartile range, *MSKCC* Memorial Sloan Kettering Cancer Center, *non-dHGP* non-desmoplastic type histopathological growth pattern, *pT-stage* tumor-stage derived from pathology report

**Table 2:** Uni- and multivariable Cox regression analysis for overall survival in non-dHGP patients (not pretreated) (n = 489)

Covariate	Univariable			Multivariable		
	HR	95% CI	P value	HR	95% CI	P value
Non-dHGP						
Age at resection	1.02	1.01–1.03	0.006	1.02	1.01–1.03	0.006
Right–sided primary tuimor	1.27	0.97–1.66	0.08	1.36	1.03–1.80	0.03
Clinical risk score (3–5)	1.72	1.34–2.23	< 0.001	1.85	1.43–2.41	< 0.001
R1 resection	1.37	1.00–1.88	0.05	1.21	0.86–1.70	0.28
Adjuvant CTx	0.53	0.39–0.73	< 0.001	0.52	0.37–0.73	< 0.001

*CI* confidence interval, *CTx* chemotherapy, *non-dHGP* non-desmoplastic type histopathological growth pattern, *HR* hazard ratio, *R1 resection* positive resection margin

The median follow-up time for survivors was 83.0 months (IQR 51–118 months), and 720 patients (54.8%) died during follow-up. The 5-year OS for patients from MSKCC not treated with adjuvant CTx was 46.9% (95% CI 38.8%–56.7%) compared to 46.5% (95% CI 41.1%–52.6%) for patients from Erasmus MC (p = 0.83).

### Overall survival and HGPs

Patients with dHGP had a 5-year OS of 63.4% (95% CI 57.7%–69.7%) compared to 45.9% (95% CI 42.6%–49.5%) in patients with non-dHGP (p < 0.001) (Appendix Fig. 4). In multivariable analysis, including the whole cohort, HGP was an independent predictor for OS (adjusted HR 1.57, 95% CI 1.29–1.92, p = 0.008) (Appendix Table 3).

### Adjuvant chemotherapy and HGPs in patients without pretreatment

**Fig. 3:**
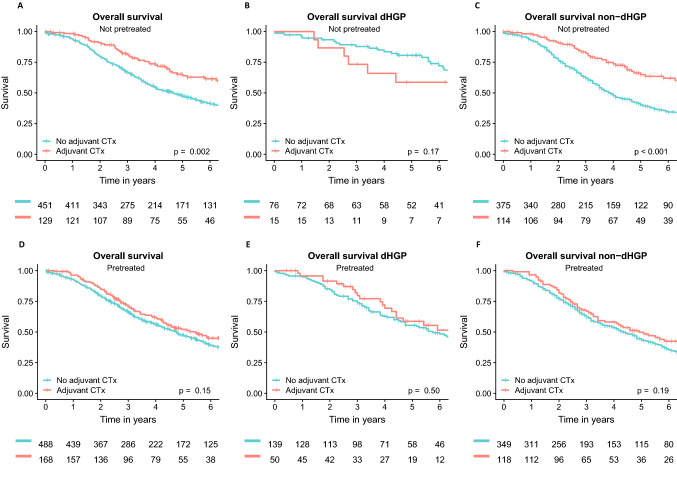
Kaplan–Meier of overall survival. Patients treated with adjuvant CTx were compared to patients not treated with adjuvant CTx in the population of patients that were not pretreated (**a**–**c**). The following populations were evaluated: **a** total patient cohort not pretreated, **b** dHGP patients not pretreated, and **c** non-dHGP patients not pretreated. Furthermore, patients treated with adjuvant CTx were compared to patients not treated with adjuvant CTx in the population of patients that were pretreated (**d**–**f**). The following populations were evaluated: **d** total patient cohort pretreated, **e** dHGP patients pretreated, and **f** non-dHGP patients pretreated

Of all 1236 patients, 580 patients (46.9%) did not receive preoperative chemotherapy. Most of these patients originated from Erasmus MC (n = 377, 65.0%). Adjuvant CTx was administered in 129 patients (21.1%) of this subgroup. Five-year OS was 65.2% (95% CI 56.7%–74.9%) in patients treated with adjuvant CTx compared to 47.5% (95% CI 42.9%–52.6%) in patients not treated with adjuvant CTx (p = 0.002) (Fig. 3a).

No difference in 5-year OS was observed in dHGP patients treated with adjuvant CTx compared to patients not treated with adjuvant CTx (p = 0.17) (Fig. 3b). A 5-year OS (Fig. 3c) of 64.9% (95% CI 55.8%–75.5%) was observed in non-dHGP patients treated with adjuvant CTx compared 40.3% (95% CI 35.3%–45.9%) in patients not treated with adjuvant CTx (p < 0.001).

In multivariable analysis (Table 2) adjuvant systemic CTx was associated with a superior OS in non-dHGP patients (adjusted HR 0.52, 95% CI 0.37–0.72, p < 0.001), but not in dHGP patients (adjusted HR 1.78, 95% CI 0.75–4.21, p = 0.19) (Appendix Table 4).

### Adjuvant systemic chemotherapy and HGPs in patients with pretreatment

A total of 656 patients (53.1%) patients received preoperative chemotherapy, of which 352 originated from MSKCC (53.7%). Adjuvant CTx was administered in 168 patients (25.6%) of patients who were pretreated prior to surgery. Five-year OS was 52.2% (95% CI 44.4%–61.3%) in patients treated with adjuvant CTx compared to 47.6% (95% CI 43.1%–52.7%) in patients not treated with adjuvant CTx (p = 0.15) (Fig. 3d).

No difference in 5-year OS was observed in dHGP and non-dHGP patients treated with adjuvant CTx compared to patients not treated with adjuvant CTx (p = 0.50 and p = 0.19) (Fig. 3e and f). In multivariable analysis adjuvant CTx was not associated with OS in dHGP patients (adjusted HR 0.83, 95% CI 0.49–1.42, p = 0.50), nor in non-dHGP patients (adjusted HR 0.96, 95% CI 0.71–1.29, p = 0.79) (Appendix Table 5).

### Disease-free survival and HGPs

A superior 5-year DFS of 35.7% was found for patients with a dHGP compared to 18.7% in patients with a non-dHGP (p < 0.001). HGP was an independent factor for DFS in multivariable analysis (adjusted HR non-dHGP 1.52, 95% CI 1.28–1.80, p < 0) (Appendix Table 6).

Superior 5-year DFS with adjuvant systemic treatment was only observed in patients with a non-dHGP that were not pretreated (20.4% versus 10.1%, p < 0.001) (Appendix Fig. 5c). This was confirmed in multivariable analysis (adjusted HR 0.71, 95% CI 0.55–0.93, p < 0.001) (Appendix Table 7 and 8).

## Discussion

This study investigates whether histopathological growth patterns predict the effect of adjuvant systemic chemotherapy after resection of CRLM. The results suggest that HGPs, that are assessed after resection of CRLM, are associated with the effectiveness of adjuvant CTx. Adjuvant CTx seemed highly effective in non-dHGP patients that were not pretreated with chemotherapy, resulting in improved OS (adjusted HR 0.52, p < 0.001) and DFS (adjusted HR 0.71, p < 0.001). In dHGP patients and in non-dHGP patients pretreated with CTx, no beneficial effect of adjuvant CTx could be demonstrated. Thereby, this study suggests that HGPs can be used to select patients for adjuvant CTx.

In order to determine the effectiveness of perioperative chemotherapy, several studies have been performed [1–5]. A large randomized trial evaluated the effectiveness of perioperative FOLFOX in patients with resectable CRLM (EORTC 40,983) [1]. Although this study was not powered on OS, and OS was not the primary endpoint of the study, no significant OS benefit was found after long-term follow-up [5]. Several non-randomized studies found that subgroups of patients may benefit from additional treatment with chemotherapy. These studies suggest that (neo-)adjuvant systemic chemotherapy might improve OS in patients at high risk of recurrence (i.e. aggressive tumor biology) [6, 7]. Post hoc analysis of the EORTC 40,983 trial demonstrated beneficial progression free survival in patients with elevated preoperative CEA levels (> 5 ng/ml) [15]. Furthermore, multiple previous studies have shown that the survival of patients with non-dHGP tumors is worse [11, 12, 16, 17]. Also, non-dHGP (and especially the replacement-type of growth) is associated with several aggressive biological characteristics such as high histological grade, lack of inflammation, and increased cancer cell motility [11, 12, 16, 17]. Therefore, the observed higher effectiveness of adjuvant CTx in patients with non-dHGP, i.e. more aggressive tumors, is in line with previous research, although validation of these findings is needed. Biological explanations of why only patients with non-dHGP appear to benefit from adjuvant CTx are lacking.

A previous study suggests that the HGPs are a strong prognostic factor in patients who are not pretreated, and in pretreated patients the prognostic value was less [10]. This observation led to the analyses of the current study. In pretreated patients HGP was not suitable to identify patients that benefit from adjuvant CTx. Previously we observed a higher proportion of dHGP (30% vs 19%, p < 0.001) after preoperative chemotherapy, suggesting a potential conversion to dHGP after pretreatment [10]. All in all, we believe that preoperative chemotherapy importantly changes HGPs. This could very well explain why the effect of HGPs on the effectiveness of adjuvant chemotherapy could only be demonstrated in those who were not pre-treated with chemotherapy.

Remarkably, we found that adjuvant CTx was not beneficial at all in pretreated patients. This observation was independent for the HGP type. Similar observations were reported in previous studies, suggesting that pre- and postoperative chemotherapy is not superior to pre- or postoperative chemotherapy alone [18, 19]. Explanations for this observation remain hypothetical, especially in the field of metastasized colorectal cancer. In colorectal cancer, it has been suggested that adjuvant chemotherapeutical regimes of only 3 months are as effective as 6 months [20]. This may also have been the case in the current study. Unfortunately, we could confirm this hypothesis since the number of cycles administered was unknown.

One could hypothesize that preoperative chemotherapy may be able to eliminate (extra)hepatic micrometastases. In that case, additional chemotherapy after surgery might be unnecessary. In patients that were not pretreated, additional postoperative chemotherapy may be able to eliminate the remaining micrometastatic disease. After all, it seems that timing of chemotherapy is not crucial. Chemotherapy administered at any time pre- or postoperative may be beneficial in patients with upfront resectable CRLM.

However, adjuvant administration of chemotherapy in patients with upfront resectable CRLM may have several practical advantages compared to preoperative administration of chemotherapy. First, the normal liver parenchyma is not affected by chemotherapy prior to surgery, thereby not affecting the regenerative ability of the liver after resection. Also, the HGP can be assessed unambiguously after surgery, without the toxic effects on tumor cells and normal liver parenchyma. Adjuvant chemotherapy may also adhere to expectations of patients that prefer upfront surgery without postponement surgery by preoperative chemotherapy.

It should be noticed that the cohort of the current study comprised of initially borderline and upfront resectable CRLM that were treated with preoperative chemotherapy. In case of borderline resectable CRLM, administration of preoperative chemotherapy is obvious.

The results of this study should be interpreted in the light of several limitations. Most importantly, the non-randomized retrospective nature of this study. Some unidentified factors may have accounted for an unknown heterogeneity among the groups. In addition, the majority of patients treated with adjuvant CTx originated from MSKCC (over 95% in both groups). In the Erasmus MC Cancer Institute, no standard adjuvant CTx is given, according to the national guidelines. However, as discussed, no major significant differences were found at baseline. Furthermore, 5-year OS in patients not treated with adjuvant CTx from MSKCC and Erasmus MC was not statistically significant (49.1% versus 46.4%, p = 0.65), supporting that there are no differences in patient-outcome at baseline. Another factor that could have introduced unaccounted bias is the fact that in some patients resection was combined with ablation of one or more lesions. In some patients the HGP type could be misinterpreted, however this is probably limited since our previous study demonstrated a very high concordance of > 90% between metastases (in case of multiple CRLM in one patient) [14].

This is the first study that demonstrates the predictive value of HGPs for adjuvant CTx after resection of CRLM. HGPs are an easily available, affordable and reliable method for clinicians to gather additional information. Other studies are needed to confirm our findings. Moreover, randomized controlled trials investigating the effectiveness of adjuvant CTx might consider HGPs as a stratification factor in the analysis.

In conclusion, the current study suggests that HGPs are associated with the effectiveness of adjuvant CTx after resection of CRLM. Patients with non-dHGP seem more likely to benefit from adjuvant CTx, while patients with dHGP do not. After pre-operative chemotherapy, adjuvant chemotherapy seems of no further benefit, irrespective of HGP. Clinicians may consider both the HGP and prior chemotherapy as factors to guide the decision for adjuvant CTx after resection of CRLM.

## Figures and Tables

**Fig. 4:**
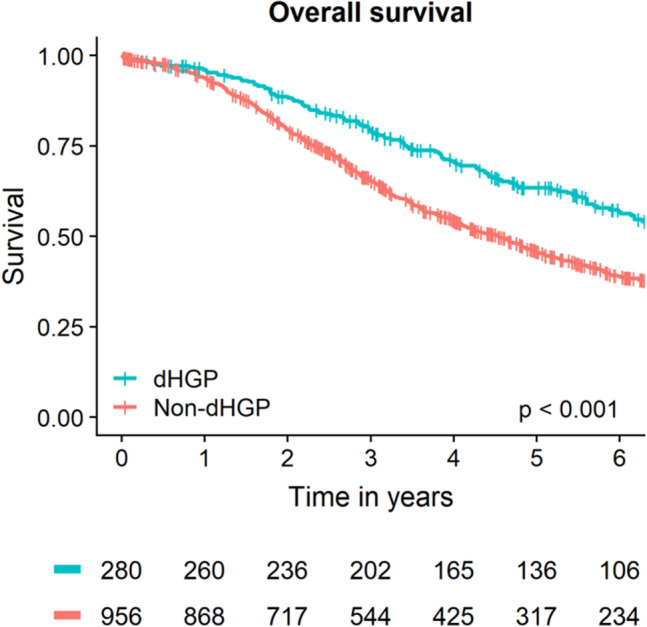
Kaplan–Meier of overall survival stratified by HGP

**Fig. 5:**
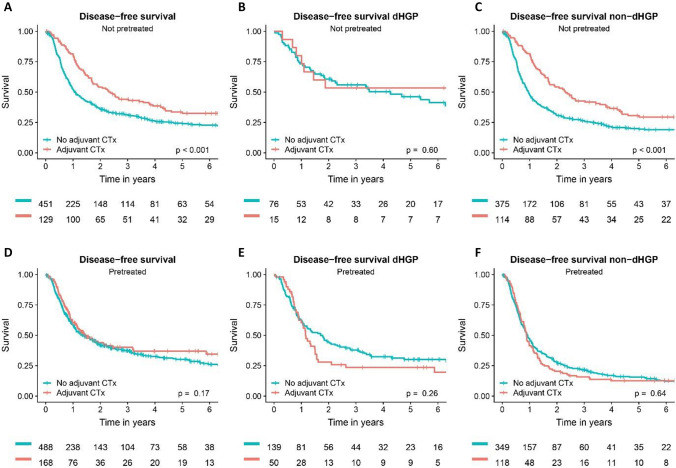
Kaplan–Meier of disease-free survival. Patients treated with adjuvant CTx were compared to patients not treated with adjuvant CTx in the population of patients that were not pretreated (**a**–**c**). The following populations were evaluated: **a** total patient cohort not pretreated, **b** dHGP patients not pretreated, and **c** non-dHGP patients not pretreated. Furthermore, patients treated with adjuvant CTx were compared to patients not treated with adjuvant CTx in the population of patients that were pretreated (**d**–**f**). The following populations were evaluated: **d** total patient cohort pretreated, **e** dHGP patients pretreated, and **f** non-dHGP patients pretreated

## Data Availability

Not generally available.

## Appendix

See Tables 3, 4, 5, 6, 7 and 8 and Figs. 4, 5.

**Table 3:** Uni- and multivariable Cox regression analysis for overall survival (n = 1236)

Covariate	Univariable			Multivariable		
	HR	95% CI	P value	HR	95% CI	P value
Age at resection	1.02	1.01–1.02	< 0.001	1.02	1.01–1.03	< 0.001
Right-sided primary tumor	1.33	1.12–1.59	0.001	1.27	1.06–1.52	0.01
Clinical risk score (3–5)	1.59	1.37–1.85	< 0.001	1.64	1.39–1.93	< 0.001
R1 resection	1.48	1.22–1.79	< 0.001	1.32	1.07–1.62	0.008
Preoperative CTx	1.11	0.96–1.28	0.17	1.12	0.95–1.32	0.17
Adjuvant CTx	1.35	1.12–1.62	0.002	0.77	0.63–0.93	< 0.001
Non-dHGP	1.54	1.28–1.86	< 0.001	1.57	1.29–1.92	0.008

*CI* confidence interval, *CTx* chemotherapy, *dHGP* desmoplastic type histopathological growth pattern, *HR* hazard ratio, *R1 resection* positive resection margin

**Table 4:** Uni- and multivariable Cox regression analysis for overall survival in dHGP patients (not pretreated) (n = 91)

Covariate	Univariable			Multivariable		
	HR	95% CI	P value	HR	95% CI	P value
dHGP						
Age at resection	1.06	1.03–1.10	< 0.001	1.04	1.00–1.08	0.03
Right-sided CRC	4.35	2.17–8.74	< 0.001	3.93	1.67–9.27	0.002
Clinical risk score (3–5)	2.42	1.13–5.18	0.02	4.01	1.72–9.37	0.001
R1 resection	1.56	0.47–5.12	0.47	2.23	0.50–9.95	0.29
Adjuvant CTx	1.66	0.78–3.57	0.19	1.78	0.75–4.21	0.19

*CI* confidence interval, *CTx* chemotherapy, *dHGP* desmoplastic type histopathological growth pattern, *HR* hazard ratio, *R1 resection* positive resection margin

**Table 5:** Uni- and multivariable Cox regression analysis for overall survival in dHGP and non-dHGP patients (pretreated) (dHGP: n = 489; non-dHGP: n = 467)

Covariate	Univariable			Multivariable		
	HR	95% CI	P value	HR	95% CI	P value
dHGP						
Age at resection	1.01	0.99–1.03	0.19	1.02	1.00–1.04	0.10
Right-sided CRC	1.21	0.73–1.99	0.46	1.17	0.70–1.95	0.56
Clinical risk score (3–5)	1.22	0.80–1.86	0.35	1.39	0.89–2.16	0.15
R1 resection	1.15	0.64–2.07	0.64	1.21	0.65–2.25	0.54
Adjuvant CTx	0.85	0.52–1.38	0.50	0.83	0.49–1.42	0.50
Non-dHGP						
Age at resection	1.02	1.01–1.03	< 0.001	1.02	1.01–1.03	0.003
Right-sided CRC	1.96	0.90–1.58	0.22	1.09	0.82–1.47	0.55
Clinical risk score (3–5)	1.53	1.21–1.95	< 0.001	1.48	1.16–1.89	0.002
R1 resection	1.48	1.13–1.94	0.005	1.38	1.04–1.85	0.03
Adjuvant CTx	0.83	0.63–1.10	0.19	0.96	0.71–1.29	0.79

*CI* confidence interval, *CTx* chemotherapy, *dHGP* desmoplastic type histopathological growth pattern, *non-dHGP* non-desmoplastic type histopathological growth pattern, *HR* hazard ratio, *R1 resection* positive resection margin

**Table 6:** Uni- and multivariable Cox regression analysis for disease-free survival (n = 1236)

Covariate	Univariable			Multivariable		
	HR	95% CI	P value	HR	95% CI	P value
Age at resection	1.00	0.99–1.01	0.90	1.00	1.00–1.01	0.28
Right-sided primary tumor	1.01	0.86–1.18	0.94	0.99	0.85–1.17	0.94
Clinical risk score (3–5)	1.61	1.41–1.84	< 0.001	1.54	1.34–1.77	< 0.001
R1 resection	1.41	1.19–1.68	< 0.001	1.33	1.11–1.59	0.002
Preoperative CTx	1.22	1.08–1.39	0.02	1.18	1.03–1.37	1.18
Adjuvant CTx	1.11	0.96–1.29	0.17	0.95	0.81–1.11	0.50
Non-dHGP	1.41	1.20–1.66	< 0.001	1.52	1.28–1.80	< 0.001

*CI* confidence interval, *CTx* chemotherapy, *non-dHGP* non-desmoplastic type histopathological growth pattern, *HR* hazard ratio, *R1 resection* positive resection margin

**Table 7:** Uni- and multivariable Cox regression analysis for disease-free survival in dHGP and non-dHGP patients (not pretreated)(dHGP n = 91, non-dHGP: n = 489)

Covariate	Univariable			Multivariable		
	HR	95% CI	P value	HR	95% CI	P value
dHGP						
Age at resection	1.01	0.99–1.04	0.31	1.01	0.98–1.04	0.47
Right-sided CRC	1.61	0.86–3.03	0.14	1.55	0.76–3.17	0.23
Clinical risk score (3–5)	2.26	1.46–4.44	0.02	2.62	1.29–5.34	0.008
R1 resection	2.00	0.79–5.10	0.15	2.63	0.88–7.84	0.08
Adjuvant CTx	0.62	0.40–1.72		0.91	0.41–2.01	0.82
Non-dHGP						
Age at resection	1.00	0.99–1.01	0.47	1.01	0.99–1.02	0.40
Right-sided CRC	0.94	0.74–1.20	0.61	1.00	0.77–1.28	0.98
Clinical risk score (3–5)	1.62	1029–2.04	< 0.001	1.63	1.29–2.05	< 0.001
R1 resection	1.35	1.01–1.81	0.04	1.32	0.97–1.79	0.08
Adjuvant CTx	0.68	0.53–0.87	0.002	0.71	0.55–0.93	0.01

*CI* confidence interval, *CTx* chemotherapy, *dHGP* desmoplastic type histopathological growth pattern, *non-dHGP* non-desmoplastic type histopathological growth pattern, *HR* hazard ratio, *R1 resection* positive resection margin

**Table 8:** Uni- and multivariable Cox regression analysis for disease-free in dHGP and non-dHGP patients (pretreated) (dHGP: n = 489; non-dHGP: n = 467)

Covariate	Univariable			Multivariable		
	HR	95% CI	P value	HR	95% CI	P value
dHGP						
Age at resection	1.00	0.99–1.02	0.70	1.01	0.99–1.03	0.36
Right-sided CRC	1.09	0.72–1.65	0.69	1.09	0.71–1.67	0.71
Clinical risk score (3–5)	1.46	1.02–2.10	0.04	1.50	1.03–2.19	0.03
R1 resection	1.33	0.80–2.19	0.27	1.26	0.74–2.16	0.40
Adjuvant CTx	1.17	0.80–1.72	0.42	1.20	0.80–1.81	0.38
Non-dHGP						
Age at resection	0.97	0.99–1.01	0.97	1.00	0.99–1.01	0.88
Right-sided CRC	0.98	0.77–1.25	0.87	0.94	0.73–1.22	0.66
Clinical risk score (3–5)	1.49	1.21–1.83	< 0.001	1.46	1.18–1.80	< 0.001
R1 resection	1.28	1.01–1.63	0.05	1.31	1.02–1.69	0.04
Adjuvant CTx	1.05	0.84–1.32	0.65	1.13	0.88–1.44	0.34

*CI* confidence interval, *CTx* chemotherapy, *dHGP* desmoplastic type histopathological growth pattern, *non-dHGP* non-desmoplastic type histopathological growth pattern, *HR* hazard ratio, *R1 resection* positive resection margin
